# Challenges of the Unknown: Clinical Application of Microbial Metagenomics

**DOI:** 10.1155/2015/292950

**Published:** 2015-09-14

**Authors:** Graham Rose, David J. Wooldridge, Catherine Anscombe, Edward T. Mee, Raju V. Misra, Saheer Gharbia

**Affiliations:** ^1^Genomic Research Unit, Public Health England, Microbiology Services, 61 Colindale Avenue, London NW9 5HT, UK; ^2^Division of Virology, National Institute for Biological Standards and Control, Medicines and Healthcare Products Regulatory Agency, South Mimms, Hertfordshire EN6 3QG, UK

## Abstract

Availability of fast, high throughput and low cost whole genome sequencing holds great promise within public health microbiology, with applications ranging from outbreak detection and tracking transmission events to understanding the role played by microbial communities in health and disease. Within clinical metagenomics, identifying microorganisms from a complex and host enriched background remains a central computational challenge. As proof of principle, we sequenced two metagenomic samples, a known viral mixture of 25 human pathogens and an unknown complex biological model using benchtop technology. The datasets were then analysed using a bioinformatic pipeline developed around recent fast classification methods. A targeted approach was able to detect 20 of the viruses against a background of host contamination from multiple sources and bacterial contamination. An alternative untargeted identification method was highly correlated with these classifications, and over 1,600 species were identified when applied to the complex biological model, including several species captured at over 50% genome coverage. In summary, this study demonstrates the great potential of applying metagenomics within the clinical laboratory setting and that this can be achieved using infrastructure available to nondedicated sequencing centres.

## 1. Introduction

**Figure 1:**
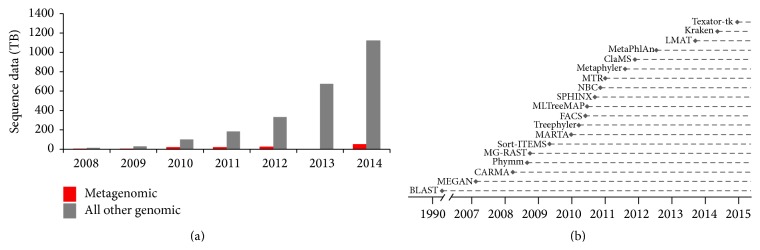
The rise of NGS data and taxonomic classification tools. (a) Data in terabytes (TB) based on NCBI SRA deposited data (data taken in January 2015). (b) A survey of metagenomic classification tools with peer reviewed publications until February 2015.

In 1995* Haemophilus influenza* was the first free-living organism to have its genome completely sequenced, which took about a year to finish the 1.8 Mb genome using conventional shotgun Sanger sequencing [1]. Contrastingly, the development of high throughput Next Generation Sequencing (NGS) platforms, with sequence outputs of now over 400,000 Mb per day, has led to a rapid increase in the number of genomes available. This is highlighted by the NCBI short read archive (SRA) passing the milestone of maintaining over 1 petabyte (PB) of whole genome sequencing (WGS) data at the end of 2014 (Figure 1(a)), with metagenomic datasets currently contributing to 49 terabytes (TB) of this. Moving from increasing sequence output, the subsequent development of benchtop sequencing platforms has enabled the transfer of NGS access from large sequencing centres to medium- and small-scale research settings. This combination of speed, capacity, and access to NGS platforms now holds great promise for public health microbiology [2, 3], as shown by recent studies on bacterial outbreaks and transmission dynamics [4–6]. Development of WGS metagenomic methods is a logical extension of NGS within the clinical setting, ultimately moving from the analysis of individual pathogens within pure cultures to all organisms from a sample, providing pathogen identification and microbial community dynamics.

The term metagenome was first coined in 1998 and was used to describe the collective genetic composition of soil microflora [7], although at this stage few genes were targeted to probe diversity among the taxa forming the microflora. Current methods used to diagnose microbial pathogens within microbial rich sites (such as the gut and respiratory tract) or normally sterile sites (such as blood or CSF) are largely based on conventional culturing of clinical samples. This consists of a battery of different growth media followed by identification, for example, by microscopy or MALDI-ToF, and then a complex series of susceptibility testing workflows are undertaken, including potential species typing [2]. The whole process can take several days to weeks and even then can result in a culture-negative result. Culture-independent methods can rapidly speed up this process to the timescale of hours through direct sequencing of the nucleic acids. While PCR assays have been designed to detect specific pathogens from clinical sample specimens [8, 9], it is the untargeted and open ended qualities of WGS metagenomics that make its application within the clinical setting advantageous, including areas such as obesity and diabetes [10, 11], as well as pathogen diagnostics [12]. However, coupling shotgun metagenomics to clinical diagnostics also presents another set of challenges, and whilst these exist at both the wet- and dry-lab side, the main bottlenecks are within data analysis and the associated storage of raw and processed data.

The computational problem of identifying pathogens within clinical metagenomics is typified by the idiom of searching for a needle in a haystack. Sample sites can be highly complex, with potential pathogens surrounded by a complex background of commensal organisms at a range of abundances in addition to host nucleic acids. Diagnostic metagenomics is one of the fastest growing fields within metagenomics, and numerous methods have been developed to help address this problem [13–31] (Figure 1(b)). These typically fall into sequence homology and to a lesser extent composition based approaches, and these have been increasing in number as the metagenomic data grows. Focusing on sequence homology, which relies on comparison to a reference genome database, methods such as MEGAN [30] use blast to bin unassembled reads or more recently employ fast short read mapping algorithms to decrease analysis times [32]. Other programs include Metaphler [33] and Sort-ITEMS [28], which are again based on blast. As an alternative to blast based methods, which can require significant computational time to search metagenomic sized datasets, recent faster methods have been based on kmer classifications such as Kraken [14] and LMAT [15]. Alternatively, the size of the comparison database can be reduced through use of smaller curated databases of gene markers, such as those employed by MetaPhIAn [16], although this provides a summary profile of the sample and does not use every read, potentially excluding useful information.

Future utility of metagenomics within the clinical setting will be dependent on the ability to detect multiple clinically relevant pathogens from a complex and host enriched background. Here we have constructed a metagenomic analysis pipeline to answer the questions: are we able to use the metagenomic approach to detect a mixture of known human viral pathogens present at range of abundances and can we capture genomes at complete coverage within an unknown and complex biological model? As part of the process of answering these questions, we describe here some of the challenges of analysing samples of unknown composition, such as the loss of sequence data through host and sample contamination. To bring the approach to the clinical laboratory all sequencing in this study was performed using benchtop technology to provide realistic sequencing depths, and the analysis pipeline was built using commodity hardware, reflecting typical infrastructure available to nondedicated sequencing centres with high throughput systems.

## 2. Materials and Methods

### 2.1. Viral Sample Nucleic Acid Extraction and Library Preparation

A reagent containing a mixed population of 25 known viruses [34] was stored at −80°C in 100 *μ*L aliquots. All aliquots were centrifuged at 3,000 ×g for 10 minutes and the supernatant was retained for generation of separate DNA and RNA libraries.

The DNA library used supernatant from one 100 *μ*L aliquot extracted using the PureLink Viral RNA/DNA kit (Life Technologies-Invitrogen) according to manufacturer's instructions, with the exception that 10 *μ*g of linear polyacrylamide (LPA) (Life Technologies-Invitrogen) was used instead of the supplied carrier RNA provided to aid with nucleic acid precipitation. Nucleic acids were eluted in 15 *μ*L of nuclease-free water. Samples were subsequently treated with 0.2 *μ*L RNase A (Epicentre) by incubating at 37°C for 15 minutes and 70°C for 20 minutes. The REPLI-g single cell kit (Qiagen) was used for amplification of DNA following the manufacturer's instructions for amplification of genomic DNA from single cells with the exception that double the volume of DNA input material was used and a 2-hour 30°C incubation performed.

For the RNA library supernatant from one 100 *μ*L aliquot was extracted using a combination of the PureLink viral RNA/DNA kit and Zymo RNA clean and concentrator kit. First the PureLink kit was used for extraction as per the manufacturer's instructions, with the addition of 5 *μ*g of LPA instead of the supplied carrier RNA. After incubation at 56°C, the sample was further purified using the Zymo RNA clean and concentrator kit as per the manufacturer's instructions using 400 *μ*L of the PureLink extract and eluted in 15 *μ*L of nuclease-free water. Samples were subsequently treated with turbo DNase (Life Technologies-Ambion) according to the recommended treatment protocol. Reverse transcription and amplification were performed according to methods described previously [35], using the Ovation RNA-Seq version 2 system (Nugen).

Amplified products were quantified using the Qubit High sensitivity kit (Life Technologies), samples were diluted to 0.2 ng/*μ*L, and DNA and cDNA libraries were prepared using the Nextera XT library preparation kit (Illumina).

### 2.2. Porcine Sample Nucleic Acid Extraction and Library Preparation

DNA extracts were prepared from 440 mg of porcine faeces. Faeces had been stored at −80°C and thawed at room temperature for 1 hour before use. To homogenise the faeces, 2 mL of 1x PBS and 1 mL of 2% 2-mercaptoethanol were added to aid in dissolution of cells and samples were placed on a rotator for 2 hours.

The homogenised faeces were transferred to a 50 mL falcon tube and centrifuged at 3,000 ×g for 10 minutes, with supernatant removed, and then 5 mL of fresh 1x PBS was added to resuspend the cells. Centrifugation and washing were repeated and the pellet was resuspended in 5 mL of 1x PBS. The homogenate was filtered through a 100 *μ*m vacuum filter (Millipore) and washed with an additional 1 mL of 1x PBS and filtered again to increase yield. The filtrate was further filtered through a 40 *μ*m vacuum filter (Millipore). Cells were centrifuged at 3,000 ×g for 10 minutes and supernatant was removed and resuspended in 1 mL 1x PBS.

Selective lysis was performed using the MolYsis selective lysis kit (Molzym) with modifications. Briefly, to 1 mL of sample, 260 *μ*L of CM buffer was added and incubated at room temperature for 15 minutes. Subsequently 250 *μ*L of DB1 buffer and 10 *μ*L of MolDNase B were added and incubated at room temperature for 15 minutes. An additional DNase treatment was performed using DNase I (Epicentre) as follows. The cells were spun at 12,000 ×g for 2 minutes and supernatant was removed. The pellet was resuspended in 100 *μ*L of a DNase I, prepared following manufacturers guidelines, and incubated at 37°C for 30 minutes. Inactivation was performed by incubation at 75°C for 15 minutes. Cells were subsequently centrifuged at 12,000 ×g for 2 minutes, supernatant was removed, and the pellet was resuspended in 1 mL of RS buffer. Cells were centrifuged at 12,000 ×g for 2 minutes and supernatant was removed. The remaining pellet was extracted using the MasterPure complete kit (Epicentre) according to manufacturer's instructions and eluted in 35 *μ*L of TE buffer.

The quality of extracted nucleic acids was determined by analysis on the tape station using the genomic DNA screen tape kit (Agilent). Average fragment size was in excess of 60 kb. Samples were prepared in duplicate, and similar to the viral sample, libraries were generated with the Nextera XT library preparation kit (Illumina) according to the manufacturer's instructions.

### 2.3. Sequencing

Prepared libraries were sized using a bioanalyser and the high sensitivity DNA kit (Agilent) and quantified using ABI Viaa7 and KAPA Illumina library quantification kit (KAPA Biosystems). Paired end (PE) sequencing was performed using an Illumina MiSeq instrument. The DNA and cDNA libraries for the viral sample were sequenced using the MiSeq Reagent Kit V2 (300 cycles) (Illumina) and the duplicate porcine libraries using the MiSeq Reagent Kit V3 (600 cycles) (Illumina), both according to manufacturer's guidelines. All sequencing data has been deposited within the EBI ENA, under the study accession number PRJEB10064 (samples ERS804108-ERS804111).

### 2.4. Quality Filtering and Removal of Host Sequences

Filtering of PE reads consisted of read inspection, trimming of adapter and poor quality bases, and finally removal of reads corresponding to host genomes. Key quality control metrics were calculated using FastQC (v0.11.2) [36], and Trimmomatic (v0.32) [37] was used to remove potential adapter contamination and poor quality reads. The adaptor database used by Trimmomatic was an in-house database of Illumina adapters, primers, and index sequences, and trimming consisted of an initial head crop of the first 15 bp, followed by iterative removal of leading and trailing bp with phred qualities < 20 and throughout the read length with mean base phred qualities < 20 in 4 bp sliding windows. Remaining sequences with lengths < 40 bp after trimming were discarded as well as orphan single end reads. Default Trimmomatic parameters were used except for those detailed above.

A mapping based approach was next used to remove host contaminant sequences. Fastq Screen (v0.4.4) (Babraham Institute) was used to map all reads to a panel of potential host genomes and all unmapped paired end reads output to new fastq files for downstream analysis. Default parameters were used within Fastq Screen, with the exception of outputting reads that did not map to any host genome using the no hits parameter and using Bowtie2 (v2.0.2) as the aligner, with all mapping parameters hard set within the Fastq Screen program. The viral sample was mapped to three host genomes: human (GRCh38), cow (Bos taurus UMD 3.1.1), and chicken (Gallus gallus-4.0), whilst the porcine sample was mapped to two host genomes: human (GRCh38) and pig (Sus scrofa 10.2). Any potential remaining vector contamination was also quantified and removed using Fastq Screen and mapping against the NCBI UniVec database (v8).

### 2.5. Species Identification

Targeted detection of the 25 viruses within the viral sample was based on the metagenomic binning tool BBSplit (v33), which uses BBMap for mapping and is part of the BBtools software suite [38]. An index of 25 respective viral genomes sequences was used as references, detailed in Table S1 in Supplementary Material available online at http://dx.doi.org/10.1155/2015/292950. Default mapping parameters were used except for the following: minratio 0.56, minhits 1, and maxindel 16000, which are equivalent to the default parameters within the BBMap. The ambig2 = split parameter was also used so that in cases where reads mapped to more than one bin, that is, more than one reference genome, these were copied into separate ambiguous bins and discarded. An in-house script was then used to parse the mapping output to generate a single mapping report file.

Ray Meta [39] (v2.3.1) was used to assemble all unmapped paired end reads from the targeted viral mapping stage. The mpi version of Ray Meta was compiled and a kmer length of 61 used based on the N50 length and number of contigs generated. Contigs <100 bp were removed. Recovery of abundance levels was achieved by reference-based mapping of the unmapped reads onto the assembled contigs using bwa mem (v.0.7.5a-r405) [40] and default parameters. Contigs were classified by blastn using the NCBI nt database (downloaded locally on 23-10-14) and subsequent blast hits filtered by a small shell script using the following criteria: percent identification ≥ 90%; *e*-value ≤1.0 × 10^−8^; bit score of ≥50. Finally, classified contigs were assigned to taxa using the Lowest Common Ancestor (LCA) algorithm as implemented within Krona Tools (v2.4) [41] and using default parameters within the ktImportBLAST and ktClassifyBLAST perl scripts, generating an html formatted Krona chart and tab formatted output, respectively. The average blast log⁡*e*-value of the contigs was used to colour the taxa within Krona.

Detection of taxa by the kmer approach used Kraken [14]. A custom database was built for Kraken using RefSeq (version 66) complete bacteria (3,471 genomes), virus (4,121 genomes), and Archaea (307) genome sequences. An additional 141 viral GenBank genomes were included to supplement viral targets used for other studies, making a total of 8,040 genomes for classification by Kraken. Default parameters were used to build the database and run the classifications, and the complete Kraken database took 2 hours to build on a server with 12 CPUs (2.7 GHz) and 132 GB of RAM, whilst classification of each sequencing dataset required 76 GB RAM. Genome coverage across select genome sequences was calculated by reference-based mapping using the above BBSplit (v33) metagenomic binning tool [38], and the genomecov command within Bedtools [42] to calculate per base genome coverage depths.

## 3. Results and Discussion

### 3.1. Datasets Generated

As proof of concept and to test the bioinformatics pipeline assembled we have sequenced and analysed two metagenomic samples selected to provide different methodological challenges using the benchtop Illumina MiSeq platform. The first dataset is a representative “knowns” mixed viral sample, containing a panel of 25 clinically relevant human viruses at a range of concentrations (abundances) as determined by qPCR and described previously [34] (Table 1). The viral sample consisted of a mixture of DNA and RNA viruses, as well as multiple serotypes, including adenovirus types 2 and 41 and parainfluenza virus types 1–4. The second dataset, a nonhuman model, was generated from porcine faeces to represent an “unknowns” sample and was expected to harbour a complex mixture of taxa at a range of abundances. The viral sample was split over two indexed libraries, as separate DNA and RNA libraries were generated to enable capture of the two viral types, which were pooled together after sequencing. The nonhuman model sample was sequenced as two technical replicates within a single MiSeq run. The technical replicates were found to be highly correlated based on genus abundance profiles using the subsequent taxonomic analysis (Pearson's *r* = 0.99) and were therefore pooled and treated as a single dataset. In total 13.5 M 150 bp and 12.7 M 300 bp paired end (PE) reads were generated for the viral panel and nonhuman model datasets, corresponding to a sequence base yield of 4.1 gigabases (Gb) and 7.6 Gb, respectively.

### 3.2. Analysis Pipeline

**Figure 2:**
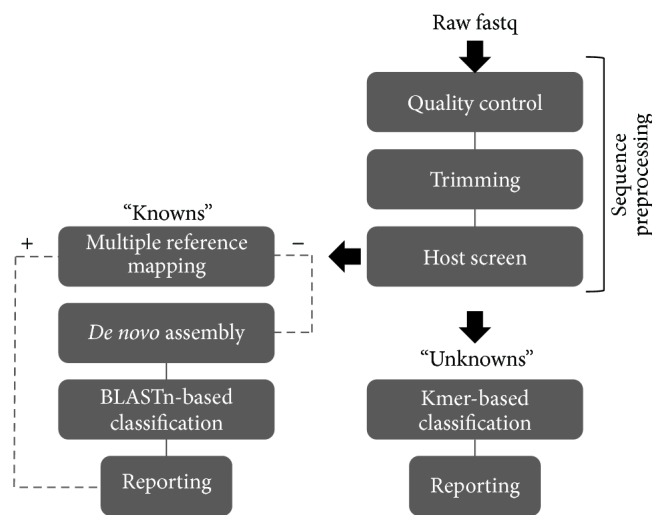
Metagenomic pipeline constructed to analyse the two datasets. Following shared sequence preprocessing steps, QC passed sequences from the known viral sample entered reference-based mapping with all unmapped reads* de novo* assembled then classified, whilst the nonhuman model sample entered a kmer-based classification pathway.

A bioinformatic pipeline was constructed to address the questions set out by this study and this was divided into two approaches (Figure 2). The “knowns” approach used the composition of the viral dataset to bin sequence reads simultaneously by mapping to reference sequences of the expected 25 viruses in a targeted approach (Table 1 and Table S1), with the aim of identifying each of the viruses from the complex viral types present, as well as from multiple host backgrounds. To further probe the rest of the sample, highlighting potential contamination, all unmapped reads were* de novo* assembled and then classified by blast. The alternative “unknowns” approach was designed for the main purpose of fast taxonomic classification of the nonhuman model sample, to identify as many taxa as possible and reveal the extent to which the genome sequences were captured.

The pipeline has three common stages, quality control, read trimming, and a host screen. Together these make up the preprocessing stages which increase the power of downstream analyses [43]. This starts by using FastQC [36] to calculate general quality control (QC) metrics, such as per base qualities, followed by Trimmomatic [37], which removes poor quality base calls and potential adapter sequences, and finally host removal by reference-based mapping using the Bowtie2 [44] aligner against a panel of expected host sequences, such as human or porcine. The targeted mapping approach has four stages. The metagenomic binning algorithm BBSplit [38] was used to simultaneously map reads to all 25 viruses, whilst ignoring ambiguous reads that map to multiple references. To enable as comprehensive as possible capture of the sample, remaining unclassified reads were assembled with RayMeta [39]. This decreased the computational burden of the later blast search against the NCBI nonredundant nt database, reducing the number of queries from millions of reads to low thousands of contigs. As a targeted mapping approach was not possible for the nonhuman model dataset, a fast classification pipeline was built around a recent kmer classification method, Kraken [14], and using a database of just over 8,000 genomes (see Section 2.5). Stages were implemented using the modular bioinformatics pipeline language, bpipe [45], which handles the initiation and overall tracking of each of the computational tasks, but with little overhead compared to other higher level pipeline tools such as Galaxy or Taverna [45, 46].

### 3.3. Species Identification within the Viral Dataset

The targeted mapping approach was used to determine if it was possible to computationally identify a complex sample of mixed virus types within one sequencing assay. Although this is unrealistic in terms of the range of viral pathogens present within a sample and used prior knowledge of the viral targets, it provides a proof of concept that can be later developed. This approach took <30 min following QC to complete mapping and reporting. Table 1 shows the number of target viral reads detected. In total 20 of the 25 viruses were identified, and these showed great variance in their relative abundances, with detection of 8 viruses based on <10,000 mapped reads, representing <0.1% of the QC passed sequence data. Interestingly, within the set of 5 undetectable viruses, 4 correlated with those that were also undetectable by qPCR (Ct > 37), whilst the remaining virus, Coronavirus 229E, had the highest recorded Ct value of the dataset (36.48).

As part of this study an alternative metagenomic classification method was used based on kmer matches to a large bacterial and viral database, and to serve as a comparison, the virus dataset was next treated as an unknown sample and entered into this pipeline. Overall the kmer-based method was highly similar to the mapping approach based on the number of reads classified to each virus (Pearson's *r* = 0.98), with 57.3% (6.5 M reads) and 43.0% (4.8 M reads) of the QC passed reads classified to the target viruses for the mapping and kmer approaches, respectively (Table 1). The whole process took just over 1 hour to report the results, with the classification of the 11.3 M input reads only taking 23 minutes (122.30 Mb/minute) and the majority of the additional time spent loading the large database into memory plus reporting the results using Krona Tools. Using this method 21 of the 25 viruses were detected (Table 1), but as before several of these were based on a very low number of classified reads, with 9 detected viruses based on <10,000 reads. Whilst these low abundance levels likely represent the low concentrations of many viruses within the sample, as reflected by the mean Ct value (31.39) (Table 1), this highlights the challenge of distinguishing very low abundance levels from a potentially complex background. Furthermore, it has recently been shown that introduction of bacterial contamination within metagenomic samples can have devastating effects on interpretation of the microbial diversity present in a sample, with contamination originating from sample preparation processes, including DNA extraction kits, reagents, and even molecular grade water [47–49].

**Figure 3:**
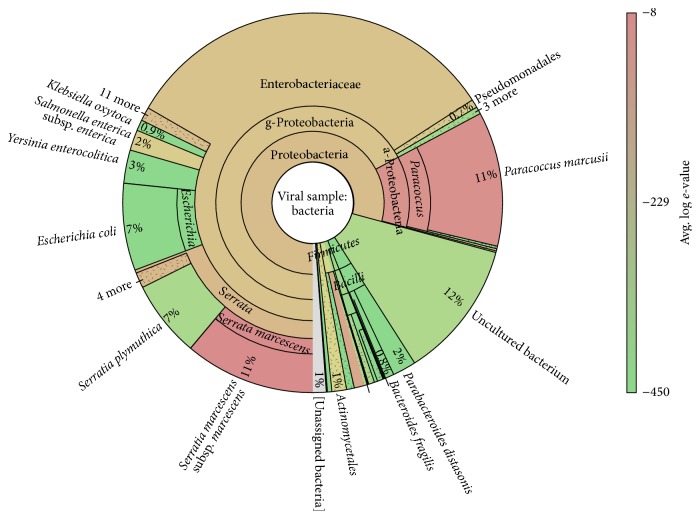
Bacterial taxa identified within the viral sample. Bacteria identified following* de novo* assembly of all unmapped reads (42.7%) from the targeted mapping approach. The Krona chart represents abundances of taxa within the 0.5 M reads corresponding to classified bacterial contigs, with abundances based on the number of reads mapping to contigs. Taxa are coloured by average contig blast log⁡*e*-value.

In an attempt to classify the origin of the remaining sequences, all 4.8 M reads from the viral dataset that were left unclassified by the targeted mapping method were entered into a further assembly based classification pipeline (Figure 2). This process took just over 7 hours to complete, generating 1,398 contigs ≥100 bases in length, with an N50 of 801 bases. However, the blast step only classified 372 of these contigs, corresponding to 31.3% (1.5 M) of the unmapped reads, with the remaining 3.3 M reads unclassified despite use of the comprehensive NCBI nt database. Over half of the reads mapping to the contigs were identified as the target viruses (0.9 M reads), likely reflecting sequence divergence from the references used in the initial targeted mapping stage. Interestingly some of the classified nontarget taxa were viral pathogens associated with the hosts used to culture the viruses, such as the identification of bovine viral diarrhoea virus, probably originating from the use of bovine serum in viral cultivation [34]. However, most of the nontarget taxa belonged to bacterial species (0.5 M reads), including the families Enterobacteriaceae and Pseudomonadaceae, such as* Escherichia coli* (Figure 3). Whilst these nontarget classifications can be ignored for the purposes of this study, such species are clearly important in the public health setting. Inclusion of appropriate no-template or no-virus/bacteria controls run in parallel with samples will likely be crucial to help define the expected level of background due to reagent contamination, sample cross-contamination, on-instrument contamination, and read misclassification.

### 3.4. Genome Capture within the Nonhuman Model

**Figure 4:**
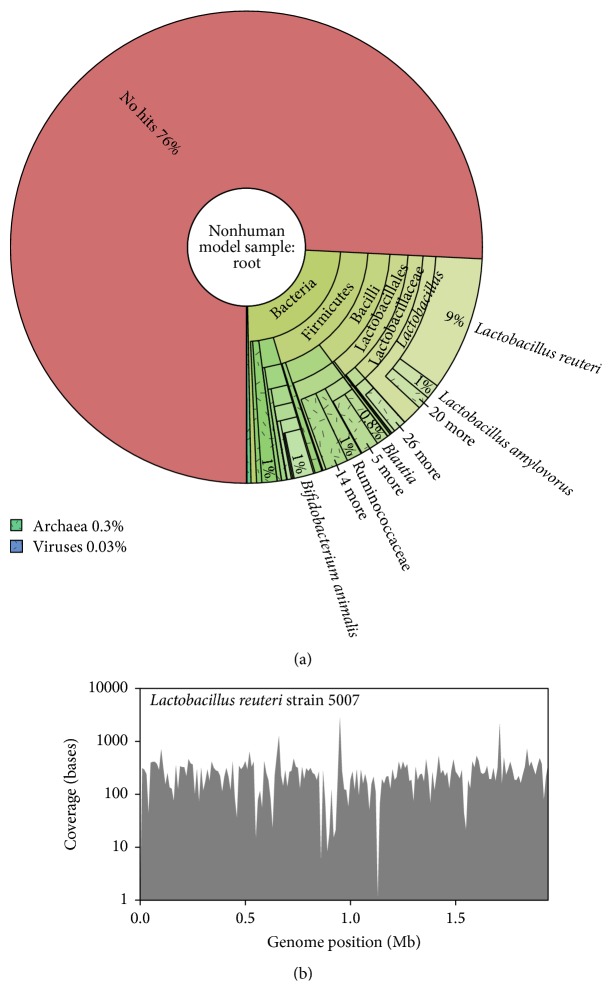
Nonhuman model sample classified using Kraken. (a) Classification of all reads represented by Krona chart. (b) Genome coverage across the 1.9 Mb genome of a reference strain from the most dominant bacterial species identified,* L. reuteri*.

We next applied the kmer classification part of the pipeline to the nonhuman model dataset. Classification of the 11.5 M QC passed reads by Kraken took 1 hour and 17 minutes (66.60 Mb/minute), and the total time to completion took <2 hours. The increased time of this analysis was due to the greater read lengths used for the nonhuman model dataset, with 300 bp reads generated instead of 150 bp for the viral panel sequencing. In total 2.8 M reads (24.2%) were classified (Figure 4(a)), and these were spread across 1,617 species, although this dropped to 146 species if only those above 0.1% abundance levels were included. The vast majority of the classified reads were identified as bacterial (98.8%). Focusing on the bacterial diversity, 5 genera accounted for 70.1% of the classified reads, consisting of* Lactobacillus*,* Bifidobacterium*,* Streptococcus*,* Blautia,* and* Ruminococcus*, all of which have been found to be highly abundant within other metagenomic studies based on porcine faecal material [50]. By far the most abundant genus was* Lactobacillus*, consisting of 23 species and 51.7% of identified reads before application of any abundance level cutoffs. A round of reference-based mapping was performed on this genus to determine the extent to which the species was captured in terms of genome coverage. As expected the highly abundant species,* Lactobacillus reuteri*, achieved the greatest complete coverage (98.6%) across the ~2 Mb genome and at 234.7-fold depth (Figure 4(b) and Table 2), with three other species achieving greater than 50% coverage. The remaining 19 species were of low abundances (<0.5%) and consequently achieved minimal genome coverage (<5%), demonstrating the need for a greater sequence output should genome capture at this abundance level be required.

Following sequencing, the millions of short reads generated proceeded through several preprocessing steps and finally taxonomic classification, all of which result in a reduction in usable read numbers. To illustrate the progressive loss of sequencing reads and fluctuations in sizes of the sequence and intermediate data, Table 3 shows the number of sequences within specific stages of the pipeline and the storage required for these. Predicted read numbers for the two samples were based on the maximum output from the sequencing chemistry kits used. These can help guide the experimental design, including the desired number of sequencing reads to capture the microbial community expected within the sample [51]. But as seen for the nonhuman model, where 50.9% of the predicted reads were obtained, effects such as low flow cell cluster density can result in lower than expected output. Preprocessing is another source of sequence loss, resulting in 2.3 M reads (16.8% of raw reads) and 1.2 M reads (9.6% of raw reads) being discarded from the viral and nonhuman model datasets, respectively (Table 3). Ultimately, 53.4% of the viral dataset and 11.2% of the nonhuman model dataset reads were used in classification of the microbial diversity. Either an increase in the number of reads generated or alternative library normalisation methods are required to provide better capture of the genome sequences within the metagenomic samples.

## 4. Conclusions

For metagenomics to be transferred from the experimental research setting and to be used for discovery of emerging pathogens and variants through use in the clinical setting, new methods are required to manage and interpret the sequence data. A key aspect of any clinical assay is definition of sensitivity to detect a given pathogen. Calculation of such a parameter within metagenomics is complicated by the fact that the pathogen of interest is not defined in advance, and the concentration of pathogen nucleic acid at which clinically relevant disease occurs may vary dramatically between different agents. It is not feasible to empirically define limits of detection using dilutions of every possible pathogen; however the use of reference materials of clinical interest is a logical starting point. Here we employed two clinically relevant sample types to demonstrate a proof of concept approach to identifying multiple viral types from a complex background of host and bacteria within a known sample and extended this out into an unknown sample, capturing the genomes of several bacterial species. Our study demonstrates that it is now possible to build a diagnostic pipeline that takes raw sequence reads and provides a comprehensive bacterial and viral identification of clinically relevant samples within a few hours and on modest commodity hardware. As is common with the application of a new method, several challenges will need to be overcome, including the waste of sequencing reads corresponding to host nucleic acids, background contamination, and improved handling of the range in species abundances to facilitate better capture of microbial diversity.

## Supplementary Material

Supplementary material contains genome details of the 25 viral references used within the targeted mapping based approach. Table S1 lists the sequence accession number, fasta header description and the different viral genome features within the panel - DNA/RNA, single/double stranded, sense/antisense.

## Figures and Tables

**Table 1:** Identification of the 25 target viruses within the viral sample using the two taxonomic classification approaches. Genome lengths based on reference genome used within the study and relative viral load shown by qPCR cycle threshold (Ct). If Ct > 37 this is shown as ND (not detectable). Percentages are based on the total reads within each dataset after preprocessing stages.

Target virus	Length (bp)	Ct	Mapping approach		Kmer approach	
			Number of reads	%	Number of reads	%
Adenovirus 2	35,937	29.71	262,395	2.3	263,857	2.3
Adenovirus 41	34,188	ND	0	0.0	0	0.0
Astrovirus	6,813	30.53	19,190	0.2	17,227	0.2
Coronavirus 229E	27,317	36.48	0	0.0	0	0.0
Coxsackievirus B4	7,397	30.72	20,706	0.2	14,422	0.1
Cytomegalovirus	230,290	28.95	580,431	5.2	584,018	5.2
Epstein-Barr virus	171,823	31.27	43,600	0.4	43,882	0.4
Herpes simplex virus 1	152,261	30.59	10,189	0.1	10,220	0.1
Herpes simplex virus 2	154,675	32.48	36,786	0.3	36,865	0.3
Influenza A H1N1	10,982	32.02	157	0.0	451	0.0
Influenza A H3N2	12,990	ND	0	0.0	4	0.0
Influenza B virus	14,452	ND	0	0.0	1	0.0
Metapneumovirus A	13,335	31.86	30,256	0.3	30,588	0.3
Norovirus GI	7,623	ND	0	0.0	0	0.0
Norovirus GII	7,535	ND	3	0.0	0	0.0
Parainfluenza virus 1	15,600	34.43	37,489	0.3	33,569	0.3
Parainfluenza virus 2	15,646	33.87	5,226	0.0	5,091	0.0
Parainfluenza virus 3	15,462	ND	309	0.0	270	0.0
Parainfluenza virus 4	17,304	31.83	9,272	0.1	8,728	0.1
Parechovirus 3	7,322	29.35	3,985,296	35.4	2,371,771	21.1
Respiratory syncytial virus A	15,158	34.33	1,530	0.0	1,551	0.0
Rhinovirus A39	7,137	31.16	13,335	0.1	13,797	0.1
Rotavirus A RVA	18,562	24.49	655	0.0	13	0.0
Sapovirus	7,429	33.37	1,455	0.0	507	0.0
Varicella-zoster virus	125,144	29.02	1,398,178	12.4	1,409,763	12.5

Total classified			6,456,450	57.3	4,846,595	43.0

**Table 2:** Genome capture of *Lactobacillus*, the most dominant genus identified within the nonhuman model dataset. Reads were mapped against a reference genome of the most abundant strain within the respective species identified.

Species identified (strain name)	Mapped reads	Bases (Mb)	Depth of coverage (bp)	Genome coverage (%)
*Lactobacillus reuteri* (5007)	983,484	457.1	234.7	98.6
*Lactobacillus johnsonii* (DPC_6026)	51,947	24.2	12.3	87.8
*Lactobacillus amylovorus* (GRL_1112)	66,840	31.7	15.3	68.9
*Lactobacillus acidophilus* (30SC )	58,678	27.3	13.2	55.4

**Table 3:** Sequence output and data storage for the two datasets. The number of sequences surviving the common preprocessing stages are shown, whilst classified sequences are based on the targeted then assembly approach within the viral dataset, and the kmer based approach within the nonhuman model dataset. Percentages based on the expected number of PE sequences generated for each sequencing chemistry kit used. Storage (in GB) consists of all fastq and intermediate files including bam and bed format files, generated throughout the analysis.

Sample	Dataset 1: viral panel			Dataset 2: nonhuman model		
	Reads within set	%	Data (GB)	Reads within set	%	Data (GB)
Predicted reads	15,000,000	—	—	25,000000	—	—
Sequenced reads	13,537,917	90.3	9.1	12,734,165	50.9	13.6
Preprocessing: trimming	12,223,513	81.5	15.8	11,520,499	46.1	24.5
Preprocessing: host screen	11,265,758	75.1		11,517,217	46.1	
Classified sequences	8,006,562	53.4	7.3	2,788,450	11.2	5.5

Total storage			32.2			43.6
